# Effect of Preoperative Antianxiety Medications on Blood Pressure and Blood Loss in Total Knee Arthroplasty: A Case-Control Study

**DOI:** 10.1155/2023/6355849

**Published:** 2023-07-08

**Authors:** Zuhdi O. Elifranji, Jihad M. Al-Ajlouni, Munther G. Al-Saber, Yazan S. Hammad, Basel A. Baniatta, Sara N. Alshoubaki, Mohammad S. Jabaiti, Ahmad M. Alkhatib, Abdelrahman M. Abu awad, Abdelrahman E. Altarazi, Aseel N. Abdin, Abdallah Al-Ani, Mohammad Ali Alshrouf

**Affiliations:** ^1^Department of Special Surgery, Division of Orthopaedics, School of Medicine, The University of Jordan, Amman 11942, Jordan; ^2^Medical Internship, Jordan University Hospital, The University of Jordan, Amman 11942, Jordan; ^3^Office of Scientific Affairs and Research, King Hussein Cancer Center, Amman 11942, Jordan

## Abstract

**Background:**

The increasing number of canceled operations in patients undergoing total knee arthroplasty (TKA) due to high blood pressure readings has put a considerable burden on surgeons. In this study, we aim to assess the effect of giving antianxiety drugs preoperatively on maintaining blood pressure (BP) and blood loss for patients undergoing TKA surgery.

**Methods:**

This retrospective case-control study included patients who underwent total knee arthroplasty and divided them into two main groups: those who had taken a 3 mg bromazepam oral tablet at the night preoperatively and the control group. The blood pressure of patients was then measured preoperatively (baseline), in the morning of surgery, in the operating room before anesthesia, and during the surgery. The percentage of measured BP was calculated by dividing the measured BP by the baseline, then multiplying by 100.

**Results:**

301 patients were included in our study: 137 received bromazepam and 164 as a control group. The ratio of systolic BP (SBP) in the morning of surgery to the baseline (percentage of morning SBP) decreased significantly in the bromazepam group compared with the controls. The ratio of SBP, in the operating room before anesthesia (percentage of preanesthesia SBP) also decreased significantly in the bromazepam group. However, the percentage of SBP in the middle of surgery did not change significantly. In addition, there was a significant difference change from the baseline in diastolic BP and mean arterial BP between the two groups in the morning of surgery, inside the theatre, and in the middle of the operation. The bromazepam group also showed a significant decrease in blood loss.

**Conclusion:**

Preoperative oral antianxiety drugs (bromazepam) helps in controlling hemodynamic changes associated with anxiety, including maintaining BP in well-controlled hypertensive and healthy patients undergoing TKA, and it plays a role in decreasing the total blood loss.

## 1. Introduction

Knee osteoarthritis (OA) is considered one of the major causes of disability, especially among elderly patients above the age of 65. Patients usually suffer from pain interfering with activities of daily living, which has a major physical, social, and psychological impact on their lives [1]. Almost 250 million individuals worldwide suffer from this disease. The ongoing degenerative process of osteoarthritis, which is irreversible, necessitates either a conservative or surgical approach, with total knee arthroplasty (TKA) being the most common [2].

TKA is considered the most effective form of treatment and the gold standard of care for symptomatic individuals with severe osteoarthritis [3]. Generally speaking, it is considered one of the most common surgeries done worldwide and the most common major orthopedic surgery performed [4]. Most patients with severe knee arthritis benefit from TKA, which helps relieve pain and increase range of motion, hence, improving the quality of life [5]. In several studies, patients reported a satisfaction level of up to 90% [6]. Furthermore, the number of primary TKR procedures is thus predicted to increase by 85% by 2030 [7].

Hypertension (HTN) is considered the most common medical issue for canceled surgeries [8]. As high as 25% of hypertensive patients undergoing any surgery actually develop perioperative elevated blood pressure [9]. In addition, nonhypertensive patients may also develop elevated systolic or diastolic readings before or during anesthesia induction, much like the “white coat syndrome” exhibited in outpatient clinics [9]. Elevated blood pressure during or after surgery, whether in hypertensive or nonhypertensive patients and can have devastating consequences such as stroke, myocardial infarction, intra- or postoperative bleeding, and even death [8, 10, 11]. This intimate relationship is proportional; higher readings mean higher risk. That is why many operations, including TKA, get postponed.

Bromazepam, a benzodiazepine, is one of the most utilized drugs in the United States [12]. This medication is used for a variety of indications, among which its anxiolytic effect is of particular concern in this study [13]. Its anxiolytic effect is assumed to be useful for maintaining blood pressure (BP) in patients undergoing TKA by decreasing stress and anxiety. Therefore, this study aims to evaluate the effect of antianxiety drugs (bromazepam) on maintaining blood pressure and decreasing blood loss in TKA. We hypothesized that oral 3 mg bromazepam on the night of the operation will help in maintaining the BP the patients undergoing TKA, and it can help in decreasing the total blood loss.

## 2. Materials and Methods

### 2.1. Study Design

A retrospective case-control study of digital and paper archives of TKA patients who underwent the procedure over a period of 3 years, from January 2018 to December 2020, at tertiary care teaching hospital was conducted. A sample size of 301 patients was included in this study with 164 in the control group and 137 in the bromazepam group. The primary outcome was to assess the ability of bromazepam to maintain the blood pressure preoperatively and the morning of the surgery inside the theatre using a ratio of measured BP compared to the baseline. The secondary outcome was to see if bromazepam could reduce the total blood loss during the operation by comparing hemoglobin levels before and after surgery on day one.

The appropriate institutional review board (IRB) of the hospital approved the proposal for this study. The Code of Ethics of the World Medical Association (Declaration of Helsinki) was followed while conducting the study. Informed written consent was obtained from the patients.

### 2.2. Study Sample and Population

Inclusion criteria included patients with diagnosed with knee OA and met the indications of TKR with primary controlled HTN or nonhypertensive. Exclusion criteria included patients who had a revision TKA, bilateral TKA, anticoagulant other than aspirin, uncontrolled HTN with more than three medications, bleeding disorder, and missing key data (age, gender, full medical history, labs, or blood pressure readings).

The patients were divided into two groups. The first group was given bromazepam 3 mg oral tablet at bedtime the night before the surgery. The second group (the control group) was a patient that underwent TKA and did not receive the medication. There were two factors that decided which patients would receive the medication. The first factor was depending on the consultant performing the procedure. There were four different experienced orthopedic surgeons who had at least 10-year experience in the field, each with a different perspective regarding the use of this drug. The second factor is the availability of the medication in the hospital at the time of the surgery.

### 2.3. Preoperative Assessment

All patients planned for the total knee replacement (TKR) were sent to the anesthesia clinic on the same day of their clinic presentation. The idea of this anesthesia clinic was to provide a preoperative fitness level and to determine whether patients needed clearance from other teams, such as cardiology or pulmonary teams. All patients got an electrocardiogram (EKG), an arterial blood gas sample, and chest X-rays in the anesthesia clinic to help determine the need for a referral.

Regarding the orthopedic team, the investigations needed are bilateral knee X-rays (standing AP and lateral views) in addition to a certain set of labs, as follows: a complete blood count (CBC), a kidney function level (KFT) with the appropriate electrolytes, an international normalized ratio (INR) (as most patients undergo spinal anesthesia), an erythrocyte sedimentation rate (ESR), a C-reactive protein (CRP) (to rule out any infection), a cross-match, and finally, other labs may be required depending on the patient's condition. Urine analysis and culture were only sent in cases where patients complained of urinary tract infection symptoms.

If the anesthesia team cleared the patient and all labs were normal, the decision to proceed with TKR was taken. Patients usually got admitted to the hospital the day before surgery in order for the medical team to address the patient's concerns or questions, monitor vital signs and glucose levels (in diabetic patients), sign the consent, and mark the proper side for the surgery. A Clexane, a low molecular weight heparin (LMWH), subcutaneous injection, was given to all patients at 8:00 PM (the night of the surgery) as part of the protocol. Patients started fasting at midnight and intravenous (IV) fluids (0.9% normal saline) were given.

### 2.4. Surgical Technique

The patient was positioned supine on the table. IV tranexamic acid and antibiotic (first- or second-generation cephalosporin) were given to the patient within 1 hour of the incision before tourniquet inflation. The patient's X-rays were displayed on the screen inside the operating room. The tourniquet was applied, along with the table's side supports. Povidone solution was used to clean the entire limb. Time-out was done before any incision is made (Zimmer, P.S. System).

A mid-line skin incision was made until the tibial tuberosity. Subcutaneous tissue dissection was done, and incision of the paratenon of the quadriceps tendon was achieved. Then, a medial parapatellar approach was taken. As most knees were varus-deformed, an adequate medial release was done at the proximal tibia surface. Extension of the knee, evertion of the patella, flexing the knee, and removal of the Hoffa fat were done in a sequential manner. The anterior cruciate ligament (ACL) and posterior cruciate ligament (PCL) were removed afterwards.

At our institution, we usually start with the tibia cut. An extramedullary guide was used for the tibia, with a proximal reference point as the medial third of the tibial tuberosity and a distal reference point as the second ray of the foot. The cut was made perpendicular to the anatomical axis of the tibia with a 7-degree posterior slope. Next, an intramedullary drill was used to gain access to the femoral canal in order to introduce the femoral intramedullary jig. It was set to 6 degrees valgus (which is equivalent to the difference between the anatomical and mechanical femur axes). The distal femur was cut by 9-10 mm (standard cut). The extension gap was then measured using a spacer. Afterwards, the flexion gap was determined using an anterior-posterior sizing guide, with the white side line as the reference point. All remaining cuts were made with appropriate protection of the medial collateral ligament (MCL) and lateral collateral ligament (LCL). Afterwards, the intercondylar notch was cut and trials were applied. After satisfactory balancing, the trials are removed, cement was applied, and the final components were in place. Irrigation and the removal of any debris were achieved. Closure of the arthrotomy was done using Vicryl (Size 2) sutures. Vicryl sutures (size 0) were used to close the subcutaneous layer, and staples or Nylon (size 3.0) were used to close the skin. The sterile compression dressing was done, and the tourniquet was deflated.

### 2.5. Postoperative Care

Patients were not allowed to ambulate on the same postoperative day (postoperative day 0). Male patients typically urinated in urinals, whereas most female patients preferred to use a Foley's catheter. On the same day, X-rays of the operated knee were taken to check for component alignment, notching, or fractures. Patients resumed prophylactic Clexane 12 hours post-op. Regarding analgesia, all patients got 4 shots of IV paracetamol (1 gram), 4 shots of intramuscular (IM) morphine (according to their weight), and 2 shots of IM nonsteroidal anti-inflammatory drugs (NSAIDs) P.R.N. The IV antibiotic coverage (1st or 2nd generation cephalosporin) remained for a total of 24 hours after the surgery.

The morning of the next day (postoperative day 1) labs were sent, including a CBC and the kidney function test. The physiotherapy team guided the ambulation process, which begins with a walker. Dressing was checked on post-op day 2.

After ensuring that the pain was well controlled, labs were normal, the dressing was dry and clean, and the patient as confident enough to ambulate on his own, most patients were discharged home on post-op day 3 or 4. Patients were discharged on oral paracetamol, proton pump inhibitors (PPIs), NSAIDs, and tramadol. Regarding the anticoagulation protocol, it was surgeon specific. Therefore, patients were given either a subcutaneous heparin injection or oral aspirin (325 mg) for a total of 6 weeks. Patients were seen in the clinic at 2 weeks (to check the wound and remove stitches or staples and check range of motion), at 6 weeks (to check for new knee X-rays and to check ROM as well), and at 3 months to check the patient's overall condition.

### 2.6. Data Collection Process

Data were collected from the hospital's oracle system. Initially, all patients who underwent total knee arthroplasty from January 2018 to December 2020 were screened for the inclusion and exclusion criteria. For the patients who met the inclusion criteria, demographics including age, gender, weight, height, and comorbidities were recorder. In addition, labs including preoperative hemoglobin level (around one day before the surgery), day one postoperative hemoglobin level, and any blood transfusion were retrieved.

Patients undergoing TKA at our hospital should routinely have a controlled blood pressure before surgery; patients with uncontrolled hypertension will be evaluated by the cardiovascular team to determine the surgical risk. The blood pressure was recorded for all the patients four times which include, one as a baseline the night before the surgery, in the morning of the surgery in the same patient room, preoperatively inside the theatre before anesthesia was administered, and during the surgery after 1 hour of anesthesia. The readings were measured using electronic or digital devices. The night and morning readings were recorded by the same device. The preoperative and intraoperative theatre readings were recorded using the same device. At the baseline, there were no patients with BP higher than 160/100 mmHg and patients in both groups received the same dose of tranexamic acid with the same protocol. There were no adverse events.

To allow the comparison between the control group and the bromazepam group with blood pressure indices, a total percentage score for each blood pressure measurement was calculated as follows: percentage of the measured blood pressure = measured blood pressure/baseline blood pressure*∗*100.

### 2.7. Statistical Analysis

Statistical analyses were made with SPSS version 29.0 (Chicago, IL, USA). We used the mean ± standard deviation to describe continuous variables. Count (frequency) to describe other nominal variables. Percentage of measured BP was calculated by dividing the measured BP to the baseline then multiplied by 100. Pearson's chi-square test was used for categorical variables. An independent sample *t*-test was used to analyze the mean differences of continuous variables at the baseline. A two-way repeated measures analysis of variance (ANOVA) was utilized to examine BP differences with (control and bromazepam) as between-subject variables and (morning BP, preanesthesia BP, midoperative BP) as within-subject variables. Statistical significance was defined as a *p* value of less than 0.05.

## 3. Results

A total of 301 patients who underwent TKA in the period from January 2018 to December 2020 were included in this study. The control group included 164 patients, while the bromazepam group included 137 patients. There were 256 (85%) women and 45 (15%) men. The mean age among the cases was 67.04 ± 7.43 and in the control group was 67.94 ± 9.01. There were no significant differences in age, sex, weight, height, diagnosis of hypertension, or the baseline hypertension before the surgery (*p* > 0.05). The demographic data about the patients and their comorbidities are summarized in Table 1.

A repeated measure ANOVA demonstrated that there is a significant time and treatment effects for all outcomes. However, a combined effect between time and treatment was not statistically significant for all outcomes. Post hoc analysis demonstrated systolic, diastolic, and mean arterial morning to the baseline BP ratio and preanesthesia to the baseline BP ratio were significantly higher for patients on controls compared to their experimental counterparts. However, only the diastolic and mean arterial middle of surgery to the baseline BP ratios were significantly different among treatment groups (all *p* < 0.05).

**Figure 1:**
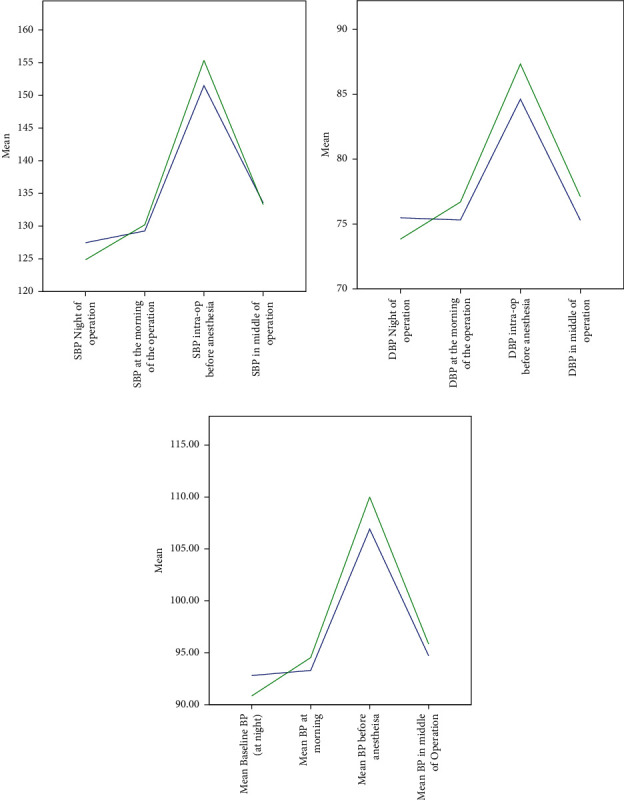
(a) Percentage change in systolic blood pressure, (b) percentage change in diastolic blood pressure, and (c) percentage change in mean arterial blood pressure in both the bromazepam group (blue line) and the control group (green line).

Moreover, Table 2 demonstrates that the differences between preanesthesia to the baseline and morning to the baseline BP ratios were statistically significant for systolic, diastolic, and mean arterial values and across both treatment groups. Similarly, the difference between preanesthesia to the baseline and middle of the surgery to the baseline BP ratios was statistically significant for systolic, diastolic, and mean arterial values and across both treatment groups. Only the systolic ratios were all significantly different from one another.  Figure 1 shows the systolic, diastolic, and mean arterial pressure changes in both the bromazepam and control groups as a percentage of the baseline at each time (morning, inside theatre, and during operation).

The hemoglobin drop on day one postoperative was significantly decreased in patients given oral bromazepam compared with the control group (*p*=0.016), with a higher mean drop of 0.24 g/dL in the hemoglobin of the control group (95% CI 0.04 to 0.43) (Table 1). However, there was no significant difference in regrades to blood transfusion in both groups (*p*=0.388).

## 4. Discussion

The purpose of this study was to evaluate the efficacy of administering antianxiety drugs prior to TKA surgery in maintaining blood pressure and decreasing total blood loss. According to our findings, we found that changes in SBP in the morning of surgery decreased significantly in the bromazepam group compared with the controls. Similarly, changes in SBP inside theatres before anesthesia also decreased significantly in the bromazepam group compared with controls. Conversely, the ratio of SBP in the middle of the surgery to the baseline did not change significantly. In addition, there was a significant difference in the changes in BP from the baseline, between the two groups in regards to the morning of the operation, in the operating room before anesthesia was given, and during the operation. Moreover, the oral bromazepam group had a lower hemoglobin drop on day one postoperative, indicating a lower blood loss.

Bromazepam is considered a member of the benzodiazepine family. They are used for various indications, including but not limited to anxiety management, insomnia, and panic attacks [14]. It is considered an intermediate-acting benzodiazepine that works on GABAA receptors to exert its inhibitory effects on the body [15]. This will lead to a reduction in the level of anxiety, which is the purpose under investigation in this study [16].

In a meta-analysis conducted by Pan et al., using data from both cross-sectional and prospective studies, it was found that there is a significant correlation and a direct association between anxiety and hypertension [17]. The mechanism by which anxiety affects blood pressure is complex, including systemic vascular resistance, plasma renin activity, sympathetic activity, and blood lipids in general. In addition, the “white coat” effect that results from anxiety is another notable example [18, 19]. Moreover, anxiety interacts with the renin-angiotensin system, increasing the level of angiotensin II [20, 21], and some studies show that patients suffering from anxiety exhibit physiological signs of sympathetic activation, as it can strongly stimulate sympathetic nervous outflow and the vasovagal reflex [22, 23]. Therefore, anxiety can activate the sympathetic nervous system, increasing cardiac output, constricting blood vessels, and raising arterial blood pressure [24].

The rate of canceled or postponed TKA surgeries due to perioperative elevated blood pressure has caught the attention of orthopedic surgeons at the hospital. Postponed surgeries mean jamming the TKA schedules, which would affect both the physical and mental status of physicians and patients. Although elevated blood pressure is considered the most common avoidable medical problem for canceling surgeries, there are still no current guidelines which would illustrate the cutoff values upon which surgeries get called off [25]. That is why the current investigation of the anxiolytic effect of bromazepam on blood pressure in those patients may be the resolution one might seek to help in controlling hemodynamic changes associated with anxiety, including maintaining BP.

Regarding the use of benzodiazepines as a blood pressure lowering agent, the literature demonstrates this fact on multiple occasions. In a retrospective study, Mendelson et al. demonstrated that the chronic use of benzodiazepines is related to a reduction in both systolic and diastolic blood pressure in individuals aged 60 and older [26]. In a recent randomised, double-blind, placebo-controlled study, Watanabe et al. concluded that using midazolam, another benzodiazepine, in a low-dose protocol would decrease blood pressure readings in patients undergoing dental procedures [27]. Gupta et al. conducted a randomized controlled study that demonstrated the benzodiazepines decrease and the sympathetic tone and blood pressure values in patients undergoing surgery when used for preoperative sedation [28]. On the other hand, nonpharmacologic therapies have proven to be quite effective, as Berbel et al. showed that listening to music prior to surgery is as effective as sedatives in lowering blood pressure measurements, heart rate, and overall anxiety level [29].

Although benzodiazepines are primarily used as an anxiolytic and hypnotic, they also have myorelaxant and vasodilatory effects [30]. Benzodiazepines have an antihypertensive effect, which could be explained by increasing the inhibitory effect of gamma-aminobutyric acid (GABA) in the CNS by binding to GABAA receptor subunits and increasing GABA ligand-receptor affinity [31]. GABA is an important neurotransmitter that is involved in many biological activities, including antihypertension, antianxiety, analgesia, and regulation of cardiovascular activity [32, 33]. Also, benzodiazepines can bind to the 18 kDa translocator protein (TPSO), formerly known as the peripheral-type benzodiazepine receptor, in addition to the GABAA receptor in the CNS. The TPSO is primarily found in mitochondria, where it is directly involved in the process of steroidogenesis [34], and as such, it plays a role in a variety of cellular functions (mitochondrial respiration, stress response, voltage-dependent calcium channel regulation, and microglia activation) and events (apoptosis, proliferation, and inflammation) [35]. TPSO is also abundant in cardiovascular tissues, where it is involved in the myocardial response to ischemia and may explain benzodiazepines' endothelium-independent vasodilatory effect [36].

The main strength of our study is that it shows that bromazepam, a commonly prescribed anxiolytic, is also associated with a reduction in the blood pressure when given to patients having total knee replacement surgery. The findings of this research have the potential to pave the way for reductions in the number of canceled procedures in the future. It also demonstrates the potential additional effect of reducing the amount of blood lost during the operation.

Limitations of this study include, first, that it was done retrospectively with patients' data collected from their archives. A better model would be a prospective randomized clinical trial. Second, the exclusion of the “amount of intraoperative blood loss” and the “duration of the surgery” from the data, as both proved to be inaccurately and irresponsibly documented in the original surgeries. This has led to relying solely on the preoperative and postoperative hemoglobin levels to determine the possible effect of decreasing blood loss during the operation. For a more accurate assessment, well-designed randomized controlled trials are required for better evaluation.

## 5. Conclusion

The result of this study provides preliminary evidence that oral bromazepam taken the night before surgery helps to maintain blood pressure in well-controlled hypertensive and healthy patients undergoing total knee arthroplasty, and it plays a positive role in decreasing total blood loss. A well-designed prospective randomized controlled trial is required for better understanding of the effect of antianxiety medications on blood pressure and blood loss in total knee arthroplasty patients.

## Figures and Tables

**Table 1:** Demographics of the patients and their comorbidities (*n* = 164 in the control group and *n* = 137 in the bromazepam group).

Variables	Control group		Bromazepam group		*p* value
	Mean ± SD	Count (%)	Mean ± SD	Count (%)	
*Gender*					0.258
Males		28 (17.1)		17 (12.4)	
Females		136 (82.9)		120 (87.6)	

Age (years)	67.94 ± 9.01		67.04 ± 7.43		0.346

Weight (kg)	85.55 ± 19.13		85.41 ± 14.51		0.941

Height (m)	160.04 ± 8.54		161.47 ± 6.30		0.105

Diagnosed with HTN, yes		106 (64.4)		101 (73.7)	0.090

Diagnosed with DM, yes		47 (40.2)		56 (40.9)	0.026

HB drop (g/dL)	1.65 ± 0.95		1.41 ± 0.75		0.016

Blood transfusion, yes		18 (11.0)		11 (8.0)	0.388

SD, standard deviation; HTN, hypertension; DM, diabetes mellitus; and HB, hemoglobin.

**Table 2:** Two-way repeated measures analysis of variance for between treatment and time interactions.

	*F* (d*f*)	*P* value
*Systolic*		
Time	253.5 (2)	<0.001
Treatment	6.9 (1)	0.009
Time*∗*treatment	2.1 (2)	0.126
	Control	Bromazepam
Morning to baseline BP ratio^*∗*^	104.9 ± 13.2^a,b,c^	101.9 ± 12.4^a,b,c^
Preanesthesia to baseline BP ratio^*∗*^	125.6 ± 17.7^a,b,c^	119.7 ± 15.2^a,b,c^
Midoperation to baseline BP ratio	107.7 ± 17.2^a,b,c^	105.4 ± 14.9^a,b,c^

*Diastolic*		
Time	119.8 (2)	<0.001
Treatment	8.2 (1)	0.004
Time*∗*treatment	0.8 (2)	0.465
	Control	Bromazepam
Morning to baseline BP ratio^*∗*^	105.2 ± 16.4^a^	101.3 ± 17.0^a^
Preanesthesia to baseline BP ratio^*∗*^	120.4 ± 20.5^a,b^	114.1 ± 17.9^a,b^
Midoperation to baseline BP ratio^*∗*^	106.4 ± 19.6^b^	101.5 ± 17.9^b^

*Mean arterial pressure*		
Time	237.3 (2)	<0.001
Treatment	9.8 (1)	0.002
Time*∗*treatment	1.7 (2)	0.192
	Control	Bromazepam
Morning to baseline BP ratio^*∗*^	104.8 ± 12.7^a^	101.4 ± 13.0^a^
Preanesthesia to baseline BP ratio^*∗*^	122.5 ± 17.1^a,b^	116.3 ± 14.0^a,b^
Midoperation to baseline BP ratio^*∗*^	106.7 ± 16.7^b^	102.9 ± 14.5^b^

BP, blood pressure. All number as in mmHg; ^*∗*^Statistically significant differences between the control and experimental groups at *p* value less than 0.05; ^a,b,c^Statistically significant differences between within-group blood pressure ratios at *p* value less than 0.05.

## Data Availability

The data from the present research that were utilized and analyzed are accessible from the corresponding author upon reasonable request.
